# Supplementary material for: Storyboarding HIV Infected Young People’s Adherence to Antiretroviral Therapy in Lower- to Upper Middle-Income Countries: A New-Materialist Qualitative Evidence Synthesis
Source: Int J Environ Res Public Health. 2022 Sep 8;19(18):11317. doi: 10.3390/ijerph191811317 (PMC9517626; doi:10.3390/ijerph191811317)
Supplement: Supplementary file 1 [file ijerph-19-11317-s001.zip › ijerph-1783122-supplementary.pdf]

Supplementary File S1: ENTREQ Reporting Guideline

No	Item	Guide and description	Page No. in manuscript
**1**	Aim	State the research questions the synthesis addresses.	7
**2**	Synthesis methodology	Identify the synthesis methodology or theoretical framework which underpins the synthesis and describe the rationale for choice of methodology *(e.g., meta-ethnography, thematic synthesis, critical interpretive synthesis, grounded theory synthesis, realist synthesis, meta-aggregation, meta-study, framework synthesis).*	2
**3**	Approach to searching	Indicate whether the search was pre-planned (*comprehensive search strategies to seek all available studies)* or iterative (*to seek all available concepts until they theoretical saturation is achieved)*.	12
**4**	Inclusion criteria	Specify the inclusion/exclusion criteria *(e.g., in terms of population, language, year limits, type of publication, study type).*	10-12
**5**	Data sources	Describe the information sources used (e.g., *electronic databases (MEDLINE, EMBASE, CINAHL, psycINFO, Econlit), grey literature databases (digital thesis, policy reports), relevant organisational websites, experts, information specialists, generic web searches (Google Scholar) hand searching, reference lists)* and when the searches conducted; provide the rationale for using the data sources.	12
**6**	Electronic Search strategy	Describe the literature search *(e.g., provide electronic search strategies with population terms, clinical or health topic terms, experiential or social phenomena related terms, filters for qualitative research, and search limits)*.	Supplement<br><br>ary File S2
**7**	Study screening methods	Describe the process of study screening and sifting *(e.g., title, abstract and full text review, number of independent reviewers who screened studies).*	12-13
**8**	Study characteristics	Present the characteristics of the included studies *(e.g., year of publication, country, population, number of participants, data collection, methodology, analysis, research questions).*	Supplement<br><br>ary File S3
**9**	Study selection results	Identify the number of studies screened and provide reasons for study exclusion *(e.g., for comprehensive searching, provide numbers of studies screened and reasons for exclusion indicated in a figure/flowchart; for iterative searching describe reasons for study exclusion and inclusion based on modifications to the research question and/or contribution to theory development).*	18-19
**10**	Rationale for appraisal	Describe the rationale and approach used to appraise the included studies or selected findings *(e.g., assessment of conduct (validity and robustness),*	20

		assessment of reporting (transparency), assessment of content and utility of the findings).	
**11**	Appraisal items	State the tools, frameworks and criteria used to appraise the studies or selected findings *(e.g. Existing tools: CASP, QARI, COREQ, Mays and Pope* [25]*; reviewer developed tools; describe the domains assessed: research team, study design, data analysis and interpretations, reporting).*	13
**12**	Appraisal process	Indicate whether the appraisal was conducted independently by more than one reviewer and if consensus was required.	13
**13**	Appraisal results	Present results of the quality assessment and indicate which articles, if any, were weighted/excluded based on the assessment and give the rationale.	Supplement<br><br>ary File S4
**14**	Data extraction	Indicate which sections of the primary studies were analysed and how were the data extracted from the primary studies? *(e.g., all text under the headings "results /conclusions" were extracted electronically and entered into a computer software).*	13
**15**	Software	State the computer software used, if any.	13
**16**	Number of reviewers	Identify who was involved in coding and analysis.	13
**17**	Coding	Describe the process for coding of data *(e.g., line by line coding to search for concepts).*	13-17
**18**	Study comparison	Describe how were comparisons made within and across studies *(e.g., subsequent studies were coded into pre-existing concepts, and new concepts were created when deemed necessary).*	13-17
**19**	Derivation of themes	Explain whether the process of deriving the themes or constructs was inductive or deductive.	13-17
**20**	Quotations	Provide quotations from the primary studies to illustrate themes/constructs and identify whether the quotations were participant quotations of the author's interpretation.	20-32
**21**	Synthesis output	Present rich, compelling, and useful results that go beyond a summary of the primary studies (e.g., *new interpretation, models of evidence, conceptual models, analytical framework, development of a new theory or construct).*	20-32

Supplementary File S2: Systematic review search strategy

Medline PubMed

Search	Query
#21	Search (#29 AND #8 AND #12 AND #17 AND #21)
#20	Search ((("vertical transmission" OR "vertical HIV transmission" OR "perinatal transmission" OR "perinatal HIV transmission" OR "vertical infection" OR "perinatal infection" OR "congenital infection" OR "vertical transfer" OR "perinatal transfer" OR "perinatal infection"))) OR "Infectious Disease Transmission, Vertical"[Mesh]
#19	Search (#17 OR #18)
#18	Search (adolescent* OR “young adult*” OR teenager* OR teens OR “high school” OR “secondary school” OR student*)
#17	Search ("Adolescent"[Mesh] OR "Young Adult"[Mesh])
#16	Search (#12 OR #13 OR #14 OR #15)
#15	Search ("developing country"[tw] OR "developing countries"[tw] OR "developing nation"[tw] OR "developing nations"[tw] OR "developing population"[tw] OR "developing populations"[tw] OR "developing world"[tw] OR "less developed country"[tw] OR "less developed countries"[tw] OR "less developed nation"[tw] OR "less developed nations"[tw] OR "less developed population"[tw] OR "less developed populations"[tw] OR "less developed world"[tw] OR "lesser developed country"[tw] OR "lesser developed countries"[tw] OR "lesser developed nation"[tw] OR "lesser developed nations"[tw] OR "lesser developed population"[tw] OR "lesser developed populations"[tw] OR "lesser developed world"[tw] OR "under developed country"[tw] OR "under developed countries"[tw] OR "under developed nation"[tw] OR "under developed nations"[tw] OR "under developed population"[tw] OR "under developed populations"[tw] OR "under developed world"[tw] OR "underdeveloped country"[tw] OR "underdeveloped countries"[tw] OR "underdeveloped nation"[tw] OR "underdeveloped nations"[tw] OR "underdeveloped population"[tw] OR "underdeveloped populations"[tw] OR "underdeveloped world"[tw] OR "middle income country"[tw] OR "middle income countries"[tw] OR "middle income nation"[tw] OR "middle income nations"[tw] OR "middle income population"[tw] OR "middle income populations"[tw] OR "low income country"[tw] OR "low income countries"[tw] OR "low income nation"[tw] OR "low income nations"[tw] OR "low income population"[tw] OR "low income populations"[tw] OR "lower income country"[tw] OR "lower income countries"[tw] OR "lower income nation"[tw] OR "lower income nations"[tw] OR "lower income population"[tw] OR "lower income populations"[tw] OR "underserved country"[tw] OR "underserved countries"[tw] OR "underserved nation"[tw] OR "underserved nations"[tw] OR "underserved population"[tw] OR "underserved populations"[tw] OR "underserved world"[tw] OR "under served country"[tw] OR "under served countries"[tw] OR "under served nation"[tw] OR "under served nations"[tw] OR "under served population"[tw] OR "under served

Search	Query
	populations"[tw] OR "under served world"[tw] OR "deprived country"[tw] OR "deprived countries"[tw] OR "deprived nation"[tw] OR "deprived nations"[tw] OR "deprived population"[tw] OR "deprived populations"[tw] OR "deprived world"[tw] OR "poor country"[tw] OR "poor countries"[tw] OR "poor nation"[tw] OR "poor nations"[tw] OR "poor population"[tw] OR "poor populations"[tw] OR "poor world"[tw] OR "poorer country"[tw] OR "poorer countries"[tw] OR "poorer nation"[tw] OR "poorer nations"[tw] OR "poorer population"[tw] OR "poorer populations"[tw] OR "poorer world"[tw] OR "developing economy"[tw] OR "developing economies"[tw] OR "less developed economy"[tw] OR "less developed economies"[tw] OR "lesser developed economy"[tw] OR "lesser developed economies"[tw] OR "under developed economy"[tw] OR "under developed economies"[tw] OR "underdeveloped economy"[tw] OR "underdeveloped economies"[tw] OR "middle income economy"[tw] OR "middle income economies"[tw] OR "low income economy"[tw] OR "low income economies"[tw] OR "lower income economy"[tw] OR "lower income economies"[tw] OR "low gdp"[tw] OR "low gnp"[tw] OR "low gross domestic"[tw] OR "low gross national"[tw] OR "lower gdp"[tw] OR "lower gnp"[tw] OR "lower gross domestic"[tw] OR "lower gross national"[tw] OR lmic[tw] OR lmics[tw] OR "third world"[tw] OR "lami country"[tw] OR "lami countries"[tw] OR "transitional country"[tw] OR "transitional countries"[tw])
#14	Search (Africa[tw] OR Asia[tw] OR Caribbean[tw] OR West Indies[tw] OR South America[tw] OR Latin America[tw] OR Central America[tw] OR Afghanistan[tw] OR Albania[tw] OR Algeria[tw] OR Angola[tw] OR Antigua[tw] OR Barbuda[tw] OR Argentina[tw] OR Armenia[tw] OR Armenian[tw] OR Aruba[tw] OR Azerbaijan[tw] OR Bahrain[tw] OR Bangladesh[tw] OR Barbados[tw] OR Benin[tw] OR Byelarus[tw] OR Byelorussian[tw] OR Belarus[tw] OR Belorussian[tw] OR Belorussia[tw] OR Belize[tw] OR Bhutan[tw] OR Bolivia[tw] OR Bosnia[tw] OR Herzegovina[tw] OR Hercegovina[tw] OR Botswana[tw] OR Brasil[tw] OR Brazil[tw] OR Bulgaria[tw] OR Burkina Faso[tw] OR Burkina Fasso[tw] OR Upper Volta[tw] OR Burundi[tw] OR Urundi[tw] OR Cambodia[tw] OR Khmer Republic[tw] OR Kampuchea[tw] OR Cameroon[tw] OR Cameroons[tw] OR Cameron[tw] OR Camerons[tw] OR Cape Verde[tw] OR Central African Republic[tw] OR Chad[tw] OR Chile[tw] OR China[tw] OR Colombia[tw] OR Comoros[tw] OR Comoro Islands[tw] OR Comores[tw] OR Mayotte[tw] OR Congo[tw] OR Zaire[tw] OR Costa Rica[tw] OR Cote d'Ivoire[tw] OR Ivory Coast[tw] OR Croatia[tw] OR Cuba[tw] OR Cyprus[tw] OR Czechoslovakia[tw] OR Czech Republic[tw] OR Slovakia[tw] OR Slovak Republic[tw] OR Djibouti[tw] OR French Somaliland[tw] OR Dominica[tw] OR Dominican Republic[tw] OR East Timor[tw] OR East Timur[tw] OR Timor Leste[tw] OR Ecuador[tw] OR Egypt[tw] OR United Arab Republic[tw] OR El Salvador[tw] OR Eritrea[tw] OR Estonia[tw] OR Ethiopia[tw] OR Fiji[tw] OR Gabon[tw] OR Gabonese Republic[tw] OR Gambia[tw] OR Gaza[tw] OR Georgia Republic[tw] OR Georgian Republic[tw] OR Ghana[tw] OR Gold Coast[tw] OR Greece[tw] OR Grenada[tw] OR Guatemala[tw] OR Guinea[tw] OR Guam[tw] OR Guiana[tw] OR Guyana[tw] OR Haiti[tw] OR Honduras[tw] OR Hungary[tw] OR India[tw] OR Maldives[tw] OR Indonesia[tw] OR Iran[tw] OR Iraq[tw] OR Isle of Man[tw] OR Jamaica[tw] OR Jordan[tw] OR Kazakhstan[tw] OR Kazakh[tw] OR Kenya[tw] OR Kiribati[tw] OR Korea[tw] OR Kosovo[tw] OR Kyrgyzstan[tw] OR Kirghizia[tw] OR Kyrgyz Republic[tw] OR Kirghiz[tw] OR

Search	Query
	Kirgizstan[tw] OR "Lao PDR"[tw] OR Laos[tw] OR Latvia[tw] OR Lebanon[tw] OR Lesotho[tw] OR Basutoland[tw] OR Liberia[tw] OR Libya[tw] OR Lithuania[tw])
#13	Search (Macedonia[tw] OR Madagascar[tw] OR Malagasy Republic[tw] OR Malaysia[tw] OR Malaya[tw] OR Malay[tw] OR Sabah[tw] OR Sarawak[tw] OR Malawi[tw] OR Nyasaland[tw] OR Mali[tw] OR Malta[tw] OR Marshall Islands[tw] OR Mauritania[tw] OR Mauritius[tw] OR Agalega Islands[tw] OR Mexico[tw] OR Micronesia[tw] OR Middle East[tw] OR Moldova[tw] OR Moldovia[tw] OR Moldovian[tw] OR Mongolia[tw] OR Montenegro[tw] OR Morocco[tw] OR Ifni[tw] OR Mozambique[tw] OR Myanmar[tw] OR Myanma[tw] OR Burma[tw] OR Namibia[tw] OR Nepal[tw] OR Netherlands Antilles[tw] OR New Caledonia[tw] OR Nicaragua[tw] OR Niger[tw] OR Nigeria[tw] OR Northern Mariana Islands[tw] OR Oman[tw] OR Muscat[tw] OR Pakistan[tw] OR Palau[tw] OR Palestine[tw] OR Panama[tw] OR Paraguay[tw] OR Peru[tw] OR Philippines[tw] OR Philipines[tw] OR Phillipines[tw] OR Phillippines[tw] OR Poland[tw] OR Portugal[tw] OR Puerto Rico[tw] OR Romania[tw] OR Rumania[tw] OR Roumania[tw] OR Russia[tw] OR Russian[tw] OR Rwanda[tw] OR Ruanda[tw] OR Saint Kitts[tw] OR St Kitts[tw] OR Nevis[tw] OR Saint Lucia[tw] OR St Lucia[tw] OR Saint Vincent[tw] OR St Vincent[tw] OR Grenadines[tw] OR Samoa[tw] OR Samoan Islands[tw] OR Navigator Island[tw] OR Navigator Islands[tw] OR Sao Tome[tw] OR Saudi Arabia[tw] OR Senegal[tw] OR Serbia[tw] OR Montenegro[tw] OR Seychelles[tw] OR Sierra Leone[tw] OR Slovenia[tw] OR Sri Lanka[tw] OR Ceylon[tw] OR Solomon Islands[tw] OR Somalia[tw] OR Sudan[tw] OR Suriname[tw] OR Surinam[tw] OR Swaziland[tw] OR Syria[tw] OR Tajikistan[tw] OR Tadzhikistan[tw] OR Tadjikistan[tw] OR Tadzhik[tw] OR Tanzania[tw] OR Thailand[tw] OR Togo[tw] OR Togolese Republic[tw] OR Tonga[tw] OR Trinidad[tw] OR Tobago[tw] OR Tunisia[tw] OR Turkey[tw] OR Turkmenistan[tw] OR Turkmen[tw] OR Uganda[tw] OR Ukraine[tw] OR Uruguay[tw] OR USSR[tw] OR Soviet Union[tw] OR Union of Soviet Socialist Republics[tw] OR Uzbekistan[tw] OR Uzbek OR Vanuatu[tw] OR New Hebrides[tw] OR Venezuela[tw] OR Vietnam[tw] OR Viet Nam[tw] OR West Bank[tw] OR Yemen[tw] OR Yugoslavia[tw] OR Zambia[tw] OR Zimbabwe[tw] OR Rhodesia[tw])
#12	Search (Developing Countries[Mesh:noexp] OR Africa[Mesh:noexp] OR Africa, Northern[Mesh:noexp] OR Africa South of the Sahara[Mesh:noexp] OR Africa, Central[Mesh:noexp] OR Africa, Eastern[Mesh:noexp] OR Africa, Southern[Mesh:noexp] OR Africa, Western[Mesh:noexp] OR Asia[Mesh:noexp] OR Asia, Central[Mesh:noexp] OR Asia, Southeastern[Mesh:noexp] OR Asia, Western[Mesh:noexp] OR Caribbean Region[Mesh:noexp] OR West Indies[Mesh:noexp] OR South America[Mesh:noexp] OR Latin America[Mesh:noexp] OR Central America[Mesh:noexp] OR Afghanistan[Mesh:noexp] OR Albania[Mesh:noexp] OR Algeria[Mesh:noexp] OR American Samoa[Mesh:noexp] OR Angola[Mesh:noexp] OR "Antigua and Barbuda"[Mesh:noexp] OR Argentina[Mesh:noexp] OR Armenia[Mesh:noexp] OR Azerbaijan[Mesh:noexp] OR Bahrain[Mesh:noexp] OR Bangladesh[Mesh:noexp] OR Barbados[Mesh:noexp] OR Benin[Mesh:noexp] OR Byelarus[Mesh:noexp] OR Belize[Mesh:noexp] OR Bhutan[Mesh:noexp] OR Bolivia[Mesh:noexp] OR Bosnia-Herzegovina[Mesh:noexp] OR Botswana[Mesh:noexp] OR Brazil[Mesh:noexp] OR Bulgaria[Mesh:noexp] OR Burkina Faso[Mesh:noexp] OR Burundi[Mesh:noexp] OR Cambodia[Mesh:noexp]

Search	Query
	OR Cameroon[Mesh:noexp] OR Cape Verde[Mesh:noexp] OR Central African Republic[Mesh:noexp] OR Chad[Mesh:noexp] OR Chile[Mesh:noexp] OR China[Mesh:noexp] OR Colombia[Mesh:noexp] OR Comoros[Mesh:noexp] OR Congo[Mesh:noexp] OR Costa Rica[Mesh:noexp] OR Cote d'Ivoire[Mesh:noexp] OR Croatia[Mesh:noexp] OR Cuba[Mesh:noexp] OR Cyprus[Mesh:noexp] OR Czechoslovakia[Mesh:noexp] OR Czech Republic[Mesh:noexp] OR Slovakia[Mesh:noexp] OR Djibouti[Mesh:noexp] OR "Democratic Republic of the Congo"[Mesh:noexp] OR Dominica[Mesh:noexp] OR Dominican Republic[Mesh:noexp] OR East Timor[Mesh:noexp] OR Ecuador[Mesh:noexp] OR Egypt[Mesh:noexp] OR El Salvador[Mesh:noexp] OR Eritrea[Mesh:noexp] OR Estonia[Mesh:noexp] OR Ethiopia[Mesh:noexp] OR Fiji[Mesh:noexp] OR Gabon[Mesh:noexp] OR Gambia[Mesh:noexp] OR "Georgia (Republic)"[Mesh:noexp] OR Ghana[Mesh:noexp] OR Greece[Mesh:noexp] OR Grenada[Mesh:noexp] OR Guatemala[Mesh:noexp] OR Guinea[Mesh:noexp] OR Guinea-Bissau[Mesh:noexp] OR Guam[Mesh:noexp] OR Guyana[Mesh:noexp] OR Haiti[Mesh:noexp] OR Honduras[Mesh:noexp] OR Hungary[Mesh:noexp] OR India[Mesh:noexp] OR Indonesia[Mesh:noexp] OR Iran[Mesh:noexp] OR Iraq[Mesh:noexp] OR Jamaica[Mesh:noexp] OR Jordan[Mesh:noexp] OR Kazakhstan[Mesh:noexp] OR Kenya[Mesh:noexp] OR Korea[Mesh:noexp] OR Kosovo[Mesh:noexp] OR Kyrgyzstan[Mesh:noexp] OR Laos[Mesh:noexp] OR Latvia[Mesh:noexp] OR Lebanon[Mesh:noexp] OR Lesotho[Mesh:noexp] OR Liberia[Mesh:noexp] OR Libya[Mesh:noexp] OR Lithuania[Mesh:noexp] OR Macedonia[Mesh:noexp] OR Madagascar[Mesh:noexp] OR Malaysia[Mesh:noexp] OR Malawi[Mesh:noexp] OR Mali[Mesh:noexp] OR Malta[Mesh:noexp] OR Mauritania[Mesh:noexp] OR Mauritius[Mesh:noexp] OR Mexico[Mesh:noexp] OR Micronesia[Mesh:noexp] OR Middle East[Mesh:noexp] OR Moldova[Mesh:noexp] OR Mongolia[Mesh:noexp] OR Montenegro[Mesh:noexp] OR Morocco[Mesh:noexp] OR Mozambique[Mesh:noexp] OR Myanmar[Mesh:noexp] OR Namibia[Mesh:noexp] OR Nepal[Mesh:noexp] OR Netherlands Antilles[Mesh:noexp] OR New Caledonia[Mesh:noexp] OR Nicaragua[Mesh:noexp] OR Niger[Mesh:noexp] OR Nigeria[Mesh:noexp] OR Oman[Mesh:noexp] OR Pakistan[Mesh:noexp] OR Palau[Mesh:noexp] OR Panama[Mesh:noexp] OR Papua New Guinea[Mesh:noexp] OR Paraguay[Mesh:noexp] OR Peru[Mesh:noexp] OR Philippines[Mesh:noexp] OR Poland[Mesh:noexp] OR Portugal[Mesh:noexp] OR Puerto Rico[Mesh:noexp] OR Romania[Mesh:noexp] OR Russia[Mesh:noexp] OR "Russia (Pre-1917)"[Mesh:noexp] OR Rwanda[Mesh:noexp] OR "Saint Kitts and Nevis"[Mesh:noexp] OR Saint Lucia[Mesh:noexp] OR "Saint Vincent and the Grenadines"[Mesh:noexp] OR Samoa[Mesh:noexp] OR Saudi Arabia[Mesh:noexp] OR Senegal[Mesh:noexp] OR Serbia[Mesh:noexp] OR Montenegro[Mesh:noexp] OR Seychelles[Mesh:noexp] OR Sierra Leone[Mesh:noexp] OR Slovenia[Mesh:noexp] OR Sri Lanka[Mesh:noexp] OR Somalia[Mesh:noexp] OR South Africa[Mesh:noexp] OR Sudan[Mesh:noexp] OR Suriname[Mesh:noexp] OR Swaziland[Mesh:noexp] OR Syria[Mesh:noexp] OR Tajikistan[Mesh:noexp] OR Tanzania[Mesh:noexp] OR Thailand[Mesh:noexp] OR Togo[Mesh:noexp] OR Tonga[Mesh:noexp] OR "Trinidad and Tobago"[Mesh:noexp] OR Tunisia[Mesh:noexp] OR Turkey[Mesh:noexp] OR Turkmenistan[Mesh:noexp] OR Uganda[Mesh:noexp] OR Ukraine[Mesh:noexp] OR Uruguay[Mesh:noexp] OR USSR[Mesh:noexp] OR Uzbekistan[Mesh:noexp] OR Vanuatu[Mesh:noexp] OR

Search	Query
	Venezuela[Mesh:noexp] OR Vietnam[Mesh:noexp] OR Yemen[Mesh:noexp] OR Yugoslavia[Mesh:noexp] OR Zambia[Mesh:noexp] OR Zimbabwe[Mesh:noexp])
#11	Search (#9 OR #10)
#10	Search (qualitative OR ethno* OR phenomen* OR hermeneutic* OR "focus group*" OR "grounded theory" OR "narrative analys*" OR "lived experience*" OR "life experience*" OR "theoretical sampl*" OR purposive OR "thematic* analys*" OR "content analy*" OR "field note*" OR fieldnote* OR "field record*" OR "field stud*" OR "participant*" OR semi-structured OR semistructured OR unstructured OR in-depth OR indepth OR face-to-face OR “face to face” OR "structured categor*" OR "action research" OR audiorecord* OR taperecord* OR videorecord* OR videotap* OR audio-record* OR “audio record” OR tape-record* OR “tape record” OR video-record OR “video record” OR interview* OR "case stud*" OR “social construct”)
#9	Search (("Interviews as Topic"[Mesh] OR "Interview" [Publication Type]) OR "Qualitative Research"[Mesh] OR "Focus Groups"[Mesh])
#8	Search (#5 OR #6 OR #7)
#7	Search (Search Retention OR retain* OR "lost to follow-up" OR ("loss*" AND "follow up") OR LTFU OR “loss-to-follow-up” OR attrition OR "loss to care" OR "loss to program*" OR default* OR engage* OR disengage* OR “retention in care” OR “lost to retention”)
#6	Search ("Treatment Adherence and Compliance"[Mesh] OR "Medication Adherence"[Mesh] OR "Patient Compliance"[Mesh])
#5	Search (Adherence OR adher* OR compliance OR complian* OR comply OR complied OR noncomplian* OR non-complian* OR non-adher* OR nonadher*)
#4	Search (#1 OR #2 OR #3)
#3	Search ((Antiretroviral* OR ((anti) AND (retroviral*)) OR ARV* OR ART OR "antiretroviral therapy" OR HAART OR ((highly) AND (active) AND (antiretroviral*) AND (therap*)) OR ((anti) AND (hiv)) OR ((anti) AND (acquired immunodeficiency)) OR ((anti) AND (acquired immuno-deficiency)) OR ((anti) AND (acquired immune deficiency)) OR ((anti) AND (acquired immun*) AND (deficienc*))))
#2	Search ((antiretroviral agents [Mesh] OR antiretroviral therapy, highly active [Mesh]))
#1	Search (((((HIV OR hiv-1 OR hiv-2* OR hiv1 OR hiv2 OR hiv infect* OR human immunodeficiency virus OR human immune deficiency virus OR human immuno-deficiency virus OR human immune-deficiency virus OR ((human immun*) AND (deficiency virus)) OR acquired immunodeficiency syndromes OR acquired immune deficiency syndrome OR acquired immuno-deficiency syndrome OR acquired

Search	Query
	immune-deficiency syndrome OR ((acquired immun*) AND (deficiency syndrome)) OR HIV/AIDS)))) OR ((HIV infections [MeSH] OR HIV [MeSH])))

Supplementary File S3: Characteristics of included studies

Study ID	Country	Objective of the study	Participants and Perspectives	HIV Cascade	Design and Data Collection Method	Method of synthesis
**Abubakar 2016**(54)	Kenya	To investigate the experiences and challenges of HIV infected adolescents at the Kenyan coast. Specifically set out to answer the following research question: What are the psychosocial challenges faced by HIV infected adolescents?	YLPHIV; Key informants: Caregivers, HCW, teachers	Lived experience	Qualitative: Individually administered IDI	Framework analysis.
**Ashaba 2019**(43)	Uganda	To understand adversities facing ALWH in rural Uganda and their effects on mental health	YLHIV; Key informants: Caregivers.	Lived experience	Qualitative: IDI and FGD	Thematic content analysis
**Bakeera-Kitaka 2008**(37)	Uganda	To assess sexual and reproductive health needs and problems, as well as determinants of sexual risk-taking among young people living with HIV aged 11 21 years attending the Paediatric Infectious Disease Clinic in Kampala, Uganda.	YLHIV; Key informants: HCW	Lived experience	Grounded theory: FGD and brief anonymous self-administered questionnaires.	Grounded theory and comparative analysis
**Bernays 2015**(79)	Uganda and Zimbabwe	To describe how children11-13 years articulate 'living with' and 'growing up' with HIV and how this is shaped through their relationships with the adults around them.	YLHIV N=104; Key informants: HCW N=20 and Caregivers N= 40	Lived experience	Prospective qualitative: IDI, FGD and audio diaries.	Combined thematic and narrative analytic approach
**Bernays 2016**(77)	Uganda, Harare, and Zimbabwe	To present data on young people living with HIVs' experiences of adherence post-disclosure and, especially, their motivations for missing treatment and avoiding seeking help in the clinic for adherence issues.	YLHIV N=130.	Treatment adherence	Longitudinal qualitative: IDI	Grounded analytic approach to thematic analysis.
**Biadgilign 2009**(74)	Ethiopia	To explore barriers and facilitators to antiretroviral medication adherence among HIV-infected paediatric patients.	Key informants: HCW N=14 and Caregivers N= 12	Treatment adherence	Qualitative: IDI	Thematic content analysis
**Bikaako-Kajura 2006**(38)	Uganda	To describe contextual factors influencing adherence to daily drug regimens among paediatric and adolescent HIV/AIDS patients; to identify and describe barriers and facilitators of adherence; and	YLHIV N=42; Key informants: Caregivers N= 42	Treatment adherence	Qualitative: IDI	Thematic analysis

Study ID	Country	Objective of the study	Participants and Perspectives	HIV Cascade	Design and Data Collection Method	Method of synthesis
		to identify relevant social support mechanisms that enhance drug adherence among children.				
**Cluver 2015**(47)	South Africa	To examine the associations between adolescent knowledge of HIV-positive status and ART-adherence. It also qualitatively examines adherence-relevant experiences amongst adolescents whose HIV-positive status has been disclosed to them, as well as healthcare workers and caregivers.	YPLHIV N=43; Key informants: Caregivers N= 25	Treatment adherence	Mixed methods: Questionnaires and interviews	Grounded analytic approach to thematic analysis
**Cruz 2015**(144)	Brazil	To analyse primary socialisation of HIV-infected children and adolescents as a social process that shapes a new generation of stigmatised and vulnerable persons.	YPLHIV; Key informants: Caregivers (no sample size or demographics).	Lived experience	Qualitative: Life history narrative: Interviews, FGD, and team field diary	Thematic content analysis (hermeneutic perspective).
**de Oliveira 2012**(69)	Brazil	To understand the life projects of young adults living with HIV / AIDS infected through vertical transmission: how they deal with studies, work and building a family. To explore their experiences regarding health assistance received.	YLPHIV N=16.	Lived experience	Qualitative: Semi-structured interviews and sociodemographic questionnaire	Thematic analysis
**Denison 2015**(70)	Zambia	To explore ART adherence from the perspectives and experiences of older ALHIV (aged 15 to 18) and their adult caregivers in Zambia.	YLPHIV N=32; Key informants: Caregivers N=23	Treatment adherence	Qualitative: IDI	Thematic analysis
**Enane 2018**(4)	Botswana	To examine the full spectrum of missed opportunities for HIV care in those most vulnerable to poor outcomes.	YLPHIV N=13; Key informants: Caregivers N= 15	ART cascade	Qualitative: Interviews using semi-structured interview guides.	Thematic analysis.
**Enimil 2016**(10)	Ghana	To examine the challenges and protective factors for adolescents living with HIV within the sociocultural context of Ghana. Specific domains assessed included quality of life, physical/ medical health, psychological wellbeing, and social relationships.	YLPHIV N=20	Lived experience	Mixed methods: IDI	Grounded Theory

Study ID	Country	Objective of the study	Participants and Perspectives	HIV Cascade	Design and Data Collection Method	Method of synthesis
**Fetzer 2011**(75)	Democratic Republic of Congo	To assess barriers and facilitators of ART adherence and the reported effects of child–caregiver relationships, psychosocial support structures, perceptions of living with HIV and of the concept of being ''adherent'' to medication. Assess specific adherence experiences among children and compared adult caregiver with child perceptions of adherence to ART.	YLPHIV N=20; Key informants: Caregivers N=20	Treatment Adherence	Qualitative: IDI	Content analysis using inductive and deductive coding approaches.
**Gachanja 2015**(55)	Kenya	To understand the lived experiences of HIV-positive parents and their children during the disclosure process in Kenya.	YLPHIV N=7	Disclosure of Status and Treatment Adherence	Phenomenology: IDI	Phenomenological thematic analysis
**Galano 2016**(67)	Brazil	To explore the meanings attributed by young individuals about ''living as an adolescent with HIV'' in a group of patients that acquired the infection at birth and the elements involved with the adherence to antiretroviral treatment.	YLPHIV N=20	Lived experience	Qualitative: IDI	Thematic analysis
**Gichane 2018**(64)	Tanzania	To explore the role of caregivers in ART adherence support from the perspective of HIV-infected orphans in Tanzania to inform a mental health and adherence intervention designed specifically for ALHIV.	YLPHIV N=17	Treatment adherence	Mixed methods: IDI	Thematic analysis
**Hodes 2019**(46)	South Africa	To explore youth preferences for the multisensory components of medicine-taking.	YLPHIV N=27	Treatment adherence	Qualitative-Participatory: IDI and FGD	Thematic analysis
**Hornschuh 2017**(145)	South Africa	To explore knowledge and experiences of adherence amongst adolescents and young adults attending treatment at the Perinatal HIV Research Unit (PHRU), Soweto, South Africa.	YLPHIV N=18	Treatment adherence	Qualitative: Structured IDI and FGD	Thematic analysis
**Inzaule 2016**(39)	Uganda	To conceptualize the barriers and facilitators to long-term ART adherence in both adolescents and adults.	YLPHIV N=6; Key informants: HCW N=44	Treatment adherence	Qualitative: Semi structured IDI and FGD	Framework synthesis using thematic coding.

Study ID	Country	Objective of the study	Participants and Perspectives	HIV Cascade	Design and Data Collection Method	Method of synthesis
**Kawuma 2014**(40)	Uganda	To describe how the experience of life-long HIV and ART interplays with everyday life for young adolescents within the different care environments such as the home, in the clinic and at school.	YLPHIV N=26	Treatment adherence	Qualitative: IDI	Thematic content analysis
**Kunapareddy 2014**(56)	Kenya	To identify the key factors contributing to paediatric ART adherence in western Kenya from the perspective of perinatally infected adolescents.	YLPHIV N=23	Treatment adherence	Qualitative: Semi structured IDI, FGD, and field notes	Thematic analysis
**Li 2010**(53)	South Africa	To explore the experiences and needs of a group of adolescents living with HIV in Cape Town, South Africa.	YLPHIV N=16	Lived experience	Qualitative: Semi-structured FGD	Thematic analysis
**Loades 2018**(48)	South Africa	To explore the lived experience of fatigue among a sample of South African adolescents receiving ART.	YLPHIV N=14	Treatment adherence	Qualitative: IDI	Thematic analysis
**Luseno 2017**(57)	Kenya	To describe adolescents' experiences with HIV services at different stages of the care continuum: HIV testing, linkage to care, and retention in treatment.	YLPHIV N=21; Key informants: Caregivers N=14	Linkage and retention	Qualitative: Semi-structured IDI	Thematic framework analysis
**MacCarthy 2018**(41)	Uganda	To explore barriers to ART adherence in Uganda among adolescents (age 14–17) and youth (age 18–24), from their own perspective, complemented with insight from community members and healthcare providers, to better understand what factors especially complicate ART adherence for these groups.	YLPHIV N=25; Key informants: HCW N=16, Community Advisory Board N=9.	Treatment adherence	Qualitative: FGD	Content analysis
**Madiba 2019**(61)	Botswana	To assess the self-reported medication adherence among ALPHIV and explore structural factors that hinder or motivate adherence to ART.	YLPHIV N=30	Treatment adherence	Phenomenology: IDI	Thematic analysis
**Marukutira 2012**(63)	Botswana	To determine the factors that influence adherence to ART among adolescents who contracted HIV through vertical transmission.	YLPHIV N=8	Treatment adherence	Phenomenology: Semi-structured IDI	Thematic analysis
**Mavhu 2013**(80)	Zimbabwe	To describe the internal and external life circumstances of YPLHIV and combine it with stakeholder input and literature about the determinants of adolescent HIV adherence to strengthen and expand AFRICAAID's programme.	YLPHIV N=229; Key informants: HCW N=72	Treatment adherence	Mixed methods: Survey, IDI, FGD, and life history narratives.	Thematic analysis

Study ID	Country	Objective of the study	Participants and Perspectives	HIV Cascade	Design and Data Collection Method	Method of synthesis
**Mburu 2014**(71)	Zambia	To examine the experiences of adolescents living with HIV in Kitwe, Kalomo and Lusaka in Zambia.	YLPHIV N=229; Key informants: HCW N=38, Caregivers n=21.	Lived experience	Qualitative: Semi-structured IDI and FGD	Narrative thematic framework analysis.
**McHenry 2017**(58)	Kenya	The objective of the following study was to characterize how HIV-infected adolescents and their caregivers understood, experienced, and were impacted by HIV/AIDS-related (H/A) stigma as well as their perspectives on how to measure and intervene to reduce HIV stigma.	YLPHIV N=39; Key informants: Caregivers n=53.	Lived experience	Qualitative: FGD	Deductive coding followed by constant comparison, axial coding, and triangulation.
**Mutumba 2015**(36)	Uganda	To identify the psychosocial challenges and coping strategies among perinatal HIV- infected adolescents in Uganda.	YLPHIV N=38	Lived experience	Phenomenology: Semi-structured IDI	Thematic analysis
**Mutwa 2013**(78)	Rwanda	To better understand combination ART adherence barriers and successes in adolescents in Rwanda, we conducted a qualitative study with perinatally HIV-infected adolescents and their primary caregivers.	YLPHIV N=42	Treatment adherence	Qualitative: IDI, FGD, and role-playing sessions.	Framework analysis and thematic analysis.
**Nabukeera Barungi 2015**(42)	Uganda	To describe the level and factors associated with adherence to antiretroviral therapy (ART) as well as the 1-year retention in care among adolescents in 10 representative districts in Uganda. In addition, explored the barriers and facilitators of adherence to ART among adolescents.	YLPHIV N=267; Key informants: Caregivers n=46.	Treatment adherence and retention	Mixed methods convergent design: IDI, FGD, and Key informant interviews	Thematic analysis
**Nestadt 2018**(146)	Thailand	To qualitatively examine psychosocial needs and strengths of Thai PHIV+ adolescents and their families.	YLPHIV N=10; Key informants: HCW N=6, Caregivers n=8.	Lived experience and adherence	Qualitative: IDI and FGD	Thematic framework analysis
**Nyogea 2015**(65)	Tanzania	To explore the barriers and facilitators of adherence among children and teenagers in rural Tanzania.	YLPHIV N=36; Key informants: HCW N=2, Caregivers n=22.	Treatment adherence	Sequential explanatory mixed methods: Semi-structured IDI and FGD	Thematic content analysis

Study ID	Country	Objective of the study	Participants and Perspectives	HIV Cascade	Design and Data Collection Method	Method of synthesis
**Petersen 2010**(147)	South Africa	To examine the psychosocial challenges and protective factors for adolescents and their caregivers affected by paediatric HIV within the socio-cultural context of South Africa with the aim of using the data to inform mental health promotion and HIV prevention interventions for this population.	YLPHIV N=25; Key informants: Caregivers n=15.	Lived experience	Qualitative: Individual in-depth interviews	Thematic analysis
**Pinzon-Iregui 2017**(148)	Dominican Republic (DR)	To characterize the Dominican Republic transition (to adult HIV care) experience for youth with perinatal infections of HIV.	YLPHIV N=15; Key Informants: Caregivers N=7, HCW N=7	Transition to adult care and Adherence	Phenomenology: FGD	Grounded theory and phenomenology
**Ramaiya 2016**(66)	Tanzania	To identify salient psychosocial and mental health challenges confronted by HIV-positive youth in a resource-poor Tanzanian setting.	YLPHIV N=24	Lived experience	Mixed methods: Semi-structured IDI	Thematic analysis
**Ritchwood 2020**(149)	South Africa	To identify clinic related facilitators and barriers to treatment retention and adherence among adolescents living with HIV in Cape Town, South Africa.	YLPHIV N=20; Key Informants: Caregivers N=19, community stakeholders N=20	Adherence and Retention	Qualitative: IDI	Thematic analysis
**Rosenbaum 2017**(50)	South Africa	To develop a cultural understanding of how young people living with HIV in the Katlehong township in the Gauteng province of South Africa effectively cope with the adversities that they face and the social ecological resources that contribute to their well-being and resilience.	YLPHIV N=7; Key Informants: Caregivers N=7, mental health providers N=3	Lived experience	Transformative concurrent mixed methods design: Photovoice, FGD, photo exhibition, and questionnaire.	Thematic analysis
**Siu 2016**(150)	Uganda	To describe how, when, and with whom HIV testing and treatment decisions are made and the role of young people themselves in these endeavours.	YLHIV N=20	Testing, Linkage, and Adherence	Qualitative: IDI	Thematic content analysis
**Vreeman 2009**(60)	Kenya	To identify key factors contributing to paediatric ART adherence and to derive a culturally specific framework that describes paediatric ART adherence from the lived experiences of participants in this resource-limited setting.	Key Informants: Caregivers N=85 and other key informants n=35.	Treatment adherence	Qualitative: Key informant interviews and FGD	Grounded theory approach with constant comparative analysis

Study ID	Country	Objective of the study	Participants and Perspectives	HIV Cascade	Design and Data Collection Method	Method of synthesis
**Vreeman 2010**(59)	Kenya	To describe how a sample of paediatric caregivers in western Kenya view the disclosure of child's HIV status and how these perceptions of disclosure might impact paediatric ART adherence.	Key Informants: Caregivers N=120	Treatment adherence	Qualitative: IDI and FGD	Constant comparative analysis
**Woollett 2017**(151)	South Africa	To examine the perceptions of HIV-positive adolescents attending clinics in Johannesburg with respect to their own infection, how they were disclosed to and their mental health state using qualitative research methodology.	YLPHIV N=15	Disclosure and adherence	Qualitative: Semi-structured IDI	Thematic analysis
**Xu 2017**(152)	Thailand	To identify factors influencing adherence among perinatally infected adolescents in Thailand.	YLPHIV N=6; Key Informants: Caregivers N=6	Treatment Adherence	Qualitative: IDI	Thematic content analysis
**Yang 2018**(62)	Botswana	To explore the barriers and facilitators of adherence among adolescents identified as the best and adolescent identified as being the worst adherers.	YLPHIV N=47; Key Informants: Caregivers N=25	Treatment adherence	Qualitative: Semi-structured IDI and FDG	Thematic analysis

Focus group discussion (FGD)
Healthcare workers (HCW)
In-depth interviews (IDI)
Human Immunodeficiency Virus (HIV)
Young people living with perinatal infections of HIV (YLPHIV)
Antiretroviral Treatment (ART)

Supplementary File S4: Summary of methodological quality assessment

	1. Was there a clear statement of the aims of the research?	2. Is a qualitative methodology appropriate?	3. Was the research design appropriate to address the aims of the research?	4. Was the recruitment strategy appropriate to the aims of the research?	5. Was the data collected in a way that addressed the research issue?	6. Has the relationship between researcher and participants been considered?	7. Have ethical issues been taken into consideration?	8. Was the data analysis sufficiently rigorous?	9. Is there a clear statement of findings?	10. How valuable is the research?
Abubakar 2016	Y	Y	Y	?	Y	N	Y	Y	Y	Y
Ashaba 2019	Y	Y	Y	Y	Y	Y	Y	Y	Y	Y
Bakeera-Kitaka 2008	Y	Y	Y	Y	Y	N	?	Y	Y	Y
Bernays 2015	Y	Y	Y	Y	Y	Y	Y	Y	Y	Y
Bernays 2016	Y	Y	Y	Y	Y	Y	Y	Y	Y	Y
Biadgilign 2009	Y	Y	Y	Y	Y	?	Y	Y	Y	Y
Bikaako 2006	Y	Y	Y	Y	Y	?	Y	Y	Y	Y

Cluver 2015	Y	Y	Y	Y	Y	?	Y	N	N	Y
Cruz 2015	Y	Y	Y	N	?	N	Y	N	Y	Y
De Oliveira 2012	Y	Y	Y	Y	Y	Y	Y	N	Y	Y
Denison 2015	Y	Y	Y	Y	Y	Y	Y	Y	Y	Y
Enane 2018	Y	Y	Y	Y	Y	Y	Y	Y	Y	Y
Enimil 2016	Y	Y	?	Y	Y	?	?	N	Y	Y
Fetzer 2011	Y	Y	Y	Y	Y	Y	Y	Y	Y	Y
Gachanja 2015	Y	Y	Y	Y	Y	N	Y	Y	Y	Y
Galano 2016	Y	Y	Y	Y	Y	?	Y	Y	Y	Y
Gichane 2018	Y	Y	Y	Y	Y	N	Y	Y	Y	Y
Hodes 2019	Y	Y	Y	Y	Y	Y	Y	Y	Y	Y
Hornschuh 2017	Y	Y	Y	Y	Y	Y	Y	Y	Y	Y
Inzaule 2016	Y	Y	Y	Y	Y	N	Y	Y	Y	Y

Kawuma 2014	Y	Y	Y	Y	Y	N	Y	Y	Y	Y
Kunapareddy 2014	Y	Y	Y	Y	Y	?	Y	Y	Y	Y
Li 2010	Y	Y	Y	Y	Y	N	Y	Y	Y	Y
Loades 2018	Y	Y	?	?	Y	N	Y	?	Y	Y
Luseno 2017	Y	Y	Y	Y	Y	N	Y	Y	Y	Y
MacCarthy 2018	Y	Y	Y	Y	Y	N	Y	Y	Y	Y
Madiba 2019	Y	Y	Y	Y	Y	Y	Y	Y	Y	Y
Marukutira 2012	Y	Y	Y	Y	Y	Y	Y	Y	Y	Y
Mavhu 2013	Y	Y	Y	Y	Y	Y	Y	Y	Y	Y
Mburu 2014	Y	Y	Y	Y	Y	Y	Y	Y	Y	Y
McHenry 2017	Y	Y	Y	Y	Y	N	Y	Y	Y	Y
Mutumba 2015	Y	Y	Y	Y	Y	N	Y	Y	Y	Y
Mutwa 2013	Y	Y	Y	Y	Y	N	Y	Y	Y	Y

Nabukeera-Barungi 2015	Y	Y	Y	Y	Y	N	Y	Y	Y	Y
Nestadt 2018	Y	Y	Y	?	Y	N	Y	Y	Y	Y
Nyogea 2015	Y	Y	Y	Y	Y	N	Y	Y	Y	Y
Peterson 2010	Y	Y	Y	?	Y	N	Y	Y	Y	Y
Pinzón-Iregui 2017	Y	Y	Y	Y	Y	?	Y	Y	Y	Y
Ramaiya 2016	Y	Y	Y	Y	Y	N	Y	Y	Y	Y
Ritchwood 2020	Y	Y	Y	Y	Y	N	Y	Y	Y	Y
Rosenbaum 2017	Y	Y	Y	Y	Y	Y	Y	Y	Y	Y
Siu 2016	Y	Y	Y	Y	Y	N	Y	Y	Y	Y
Vreeman 2009	Y	Y	Y	Y	Y	N	Y	Y	Y	Y
Vreeman 2010	Y	Y	Y	Y	Y	N	Y	Y	Y	Y
Woollett 2017	Y	Y	Y	Y	Y	N	Y	Y	Y	Y
Xu 2017	Y	Y	Y	Y	Y	Y	Y	Y	Y	Y
Yang 2017	Y	Y	Y	Y	Y	N	Y	Y	Y	Y
